# A technical feasibility study on adaptation of a microsurgical robotic system to an intraoperative complication management in dental implantology: perforated Schneiderian membrane repair using Symani^®^ Surgical System

**DOI:** 10.1007/s11701-023-01721-9

**Published:** 2023-10-06

**Authors:** Henning Wieker, Cedric Hinrichs, Merle Retzlaff, Johannes Heinrich Spille, Martin Laudien, Yahya Acil, Jörg Wiltfang, Aydin Gülses

**Affiliations:** 1 Department of Oral and Maxillofacial Surgery, Christian Albrechts University, UKSH Campus Kiel, 24105 Kiel, Germany; 2 Department of ENT Surgery, Christian Albrechts University, UKSH Campus Kiel, 24105 Kiel, Germany

**Keywords:** Dentistry, Implantology, Robotic surgery, Schneiderian membrane

## Abstract

The aim of the current study was to test the technical and clinical feasibility of a robotic system and investigate its potential in the surgical repair of perforated Schneiderian membranes using an ex-vivo porcine model. Eight pig heads were operated conventionally via a surgical loop and eight pig heads with the surgical robot “Symani^®^ Surgical System” (Medical Microinstruments, Inc., Pisa, Italy). On each specimen, the Schneiderian membrane was incised over a length of 0.7 mm resembling a perforation. Operation time, the maximum sinusoidal pressure, the course of the pressure and the filling volume were measured. Additionally, adaptation of the wound edges has been detected via scanning electron microscopy. There were no significant differences for the pressure maximum (*p* = 0.528), for the time until the pressure maximum was reached (*p* = 0.528), or for the maximum filling volume (*p* = 0.674). The time needed for the suturing of the membrane via robotic surgery was significantly longer (*p* < 0.001). However, the scanning electron microscope revealed a better adaptation of the wound edges with robotic surgery. The technical feasibility of robot-assisted suturing of Schneiderian membrane laceration using the robotic system has been confirmed for the first time. No differences considering the pressure resistance compared to the conventional repair could be observed, but advantages in wound adaptation could be found with an electron microscope. Regarding the material and training costs and limited indications spectrum, robotic surgery systems still might not present financially feasible options in the daily dental practice yet.

**Supplementary Information:**

The online version contains supplementary material available at 10.1007/s11701-023-01721-9.

## Introduction

Due to the lack of sufficient bone volume secondary to the atrophy of alveolar bone and sinus pneumatization, rehabilitation of the posterior maxilla with dental implants poses a special challenge for dental professionals [1]. A well-established technique with several modifications to overcome this problem is the grafting of the maxillary sinus after the elevation of the Schneiderian membrane, which is called as sinus lift surgery. The procedure is mainly classified into crestal (internal sinus lift) and lateral (lateral sinus lift) approaches regarding the residual bone height. In cases where the residual bone height is 5 mm or less, the lateral approach, which is performed with exploration and elevation of the sinus membrane throughout a bony window, is recommended.

Despite high success rates, intraoperative complications could jeopardize the wound healing at the grafted maxillary sinus. A normal sinus membrane thickness is only 0.3–0.8 mm, and the most common intraoperative complication is the laceration of the membrane, which could negatively affect the graft's stabilization, lead to displacement and infection of the bone graft [2]. The incidence of perforations has been stated to occur with a range from 22 to 50% [3, 4]. Additionally, Nolan et al. have stated that perforated sinuses presented three times the risk of bone graft failure and six times the incidence of maxillary sinusitis compared with non-perforated sinuses [5]. Therefore, it is necessary to be aware of the various methods for avoiding or closing perforations.

In the literature, various methods have been proposed for the intraoperative management of membrane repair including suturing with resorbable materials, sealing with fibrin glue [6], the use of collagen membrane with or without combination with platelet-rich-fibrin [7] and augmentations with a buccal fat pad or laminar bone block [3]. When perforations are smaller than 5 mm, the most widely recommended treatment is the repair with collagen membrane [8], whereas moderate perforations (5–10 mm) should be managed with resorbable sutures before the collagen membrane has been placed [6]. However, the strength of the suture and the sufficient adaptation of the rupture edges play a key role in the prognosis, thus, the closure should withstand the intra-sinusoidal pressure and prevent graft particles from escaping into the sinus during the healing phase.

The technical and clinical feasibility of robot-assisted suturing in different disciplines has become the main subject of recent studies [9]. The “Symani^®^ Surgical System” (Medical Microinstruments, Pisa, Italy) is a surgical robot, designed for microsurgery as free flap reconstructions, lymphatic surgery, nerve reconstruction or replantation. The system consists of two robotic arms on which microinstruments of a few millimetres in size can be placed. A 7–20 × motion scaling is intended to enable freedom from tremors in microsurgery, the instruments guarantee seven degrees of freedom. The surgeon operates from a console consisting of a chair, two joysticks and a foot switch. The use of the system in the maxillary sinus has not been presented until now. The aim of the current study was to test the technical feasibility of the Symani Surgical System in surgical repair of perforated Schneiderian membranes using an ex-vivo porcine model and investigate its potential advantages.

## Material and methods

**Fig. 1:**
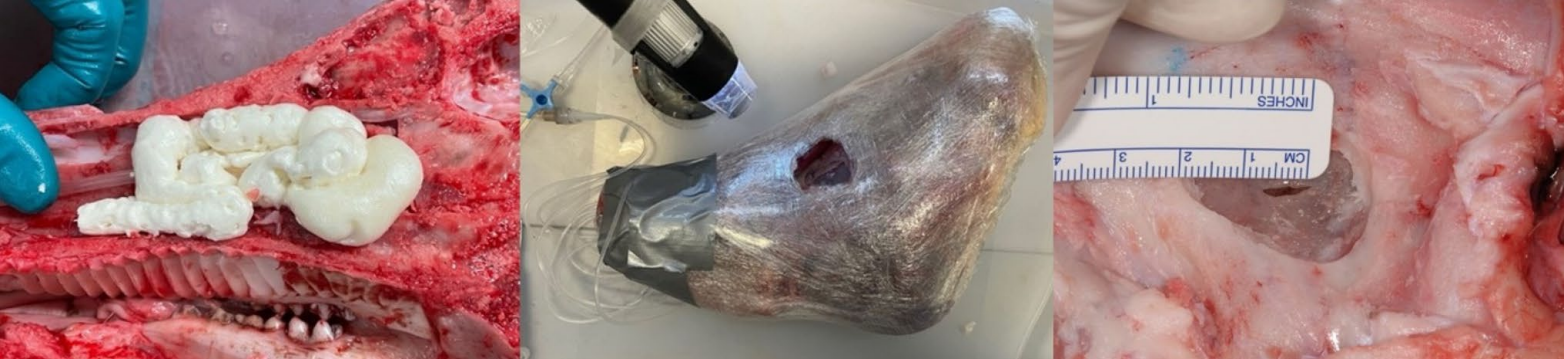
Illustration of the airtight seal with construction foam as well as plastic wrap for measuring the pressure without any leakage and the perforation of 0.7 mm

A total of 16 pig head halves were obtained commercially from a slaughterhouse in Kiel (age 16–18 months). Eight head halves were operated conventionally (free hand via a surgical loop) whereas a surgical robot “Symani® Surgical System” (Medical Microinstruments, Inc., Pisa, Italy) was used in eight specimens. In both groups, a bone window of approximately 2.5 × 1.5 × 3 cm was created on each pig head in the area of the sinus maxillaris. The Schneiderian membrane was mobilized as described by Tatum [10]. After the preparation was completed, a control measurement of the maxillary sinus pressure was taken to ensure the integrity of the mucosa. At least a pressure of 3.4 kPa was built up on the Schneiderian membrane via a perfusor and the continuity of the membrane has been approved. For this purpose, each head half was wrapped with plastic wrap for an additional seal. The bone hole of the sinus maxillaris with the Schneider's membrane was cut free for surgery and measurement. For insufflation and pressure measurement, a tube was inserted over the concha nasalis media and sealed airtight with construction foam. After that, the Schneiderian membrane was incised over a length of 0.7 mm resembling a membrane laceration. This perforation had to be closed again surgically with one of the surgical procedures (Fig. 1). To minimize the decomposition of the material during the sample preparation process for SEM and to reduce the friction when passing through the fragile membrane, a none-resorbable monofilament with very smooth surface characteristics (9–0 Prolene, Ethicon, Johnson & Johnson Medical GmbH, Norderstedt, Germany) has been selected for the repair.

**Fig. 2:**
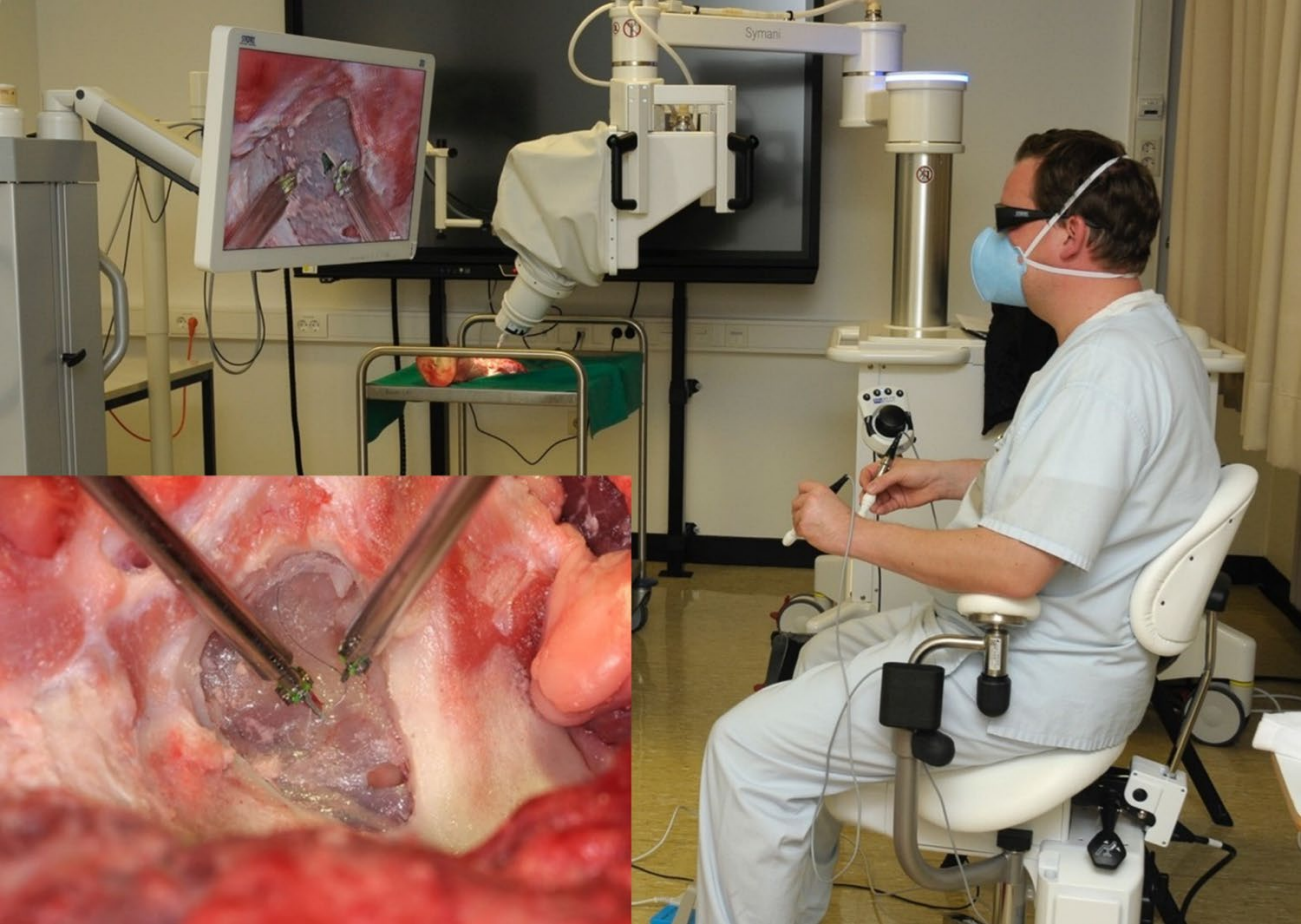
For robotic-assisted surgery, the NanoWrist Needle Holder and Dilator microinstruments with 3 mm wrists were used

The surgical closure of the perforation was achieved in both surgical procedures using 9–0 Prolene (Ethicon, Johnson & Johnson Medical GmbH, Norderstedt, Germany). All operations were performed by a single experienced surgeon for comparability. The surgeon had experience in both operation procedures. A needle holder, surgical forceps, and magnifying glasses with × 2.5 magnification were available for conventional surgery. For robotic-assisted surgery, the NanoWrist Needle Holder and Dilator microinstruments with 3 mm wrists were used (Fig. 2).

For better comparability of the two surgical procedures, a continuous suture was preferred, which had also less tendency for punctual leakages during pressure measurement. For the measurement, the tube was connected via a three-way valve to the Syramed µSP6000 perfusor (Arcomed AG Medical Systems, Zurich, Switzerland) and to the GM520 manometer (Bestone Industrial Ltd., Shenzhen, China), which is connected to a laptop for recording the pressure course For each specimen, pressure curve in the maxillary sinus was documented by the manometer, which was connected to the measuring system via the three-way valve, and the maximum pressure was determined before the suture of the Schneiderian membrane ruptured (Video 1).

In addition, the sutured laceration was examined with the scanning electron microscope XL30CP (Philips Electron Optics GmbH, Kassel, Germany). For this purpose, the membrane was first washed with phosphate-buffered saline and then dehydrated in an ascending alcohol series (2 × 10 min. 50%, 1 × 10 min. 60%, 2 × 10 min. 70%, 2 × 10 min. 90%, 3 × 10 min. 100%). After that, hexamethyldisilazane was applied for a few seconds. The membranes were post-dried in a desiccator overnight and then vaporized with 10 nm gold during sputtering (SCD 500, Bal-Tec, Balzers, Lichtenstein). The imaging was performed at a voltage of 10 kV under the scanning electron microscope.

### Statistical analysis

Statistical analyses were performed using SPSS (IBM^®^, Ehningen, Germany). The average values for the pressure maximum, the time until the pressure maximum was reached, the maximum filling volume, and the operation time were calculated (mean, standard deviation). The relation between variables was evaluated by Mann–Whitney-*U* Test. Associations were considered significant when the *p* value was < 0.05.

## Results

### Pressure measurement

**Fig. 3:**
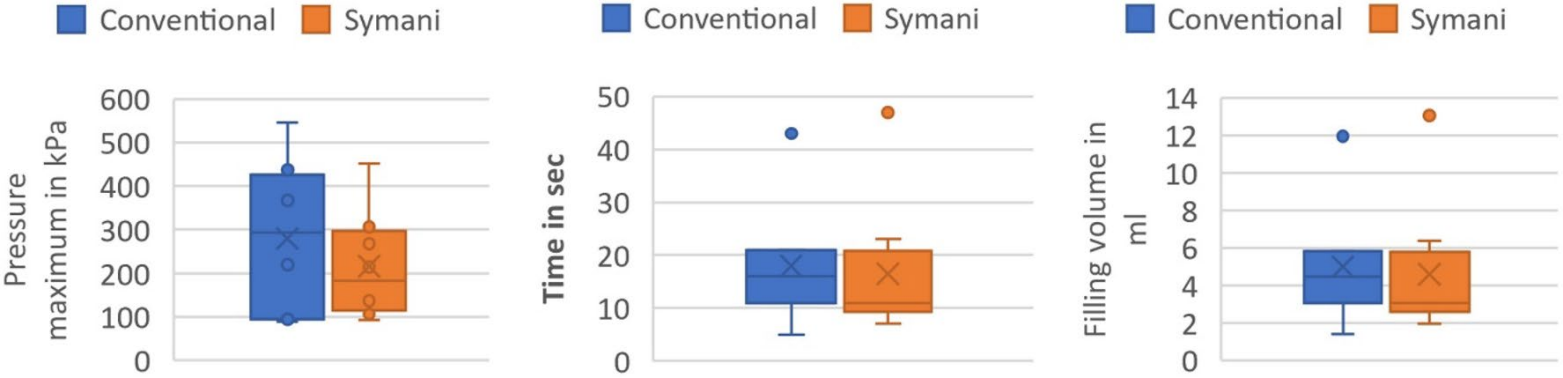
Box plots of the pressure maximum in kPa, the time in seconds until the pressure maximum was reached and the maximum filling volume in ml

 The mean values and the standard deviations for the pressure maximum, the time until the pressure maximum was reached, the filling volume, and the operation time are shown in Table 1 as well as in Fig. 3. No statistical significance was present among both groups regarding the above-mentioned pressure parameters.

**Table 1:** Imaged are the mean values and the standard deviations (SD) for both operation procedures

	Pressure maximum (Mean ± SD)	Time until pressure maximum was reached (Mean ± SD)	Filling volume (Mean ± SD)	Operation time (Mean ± SD)
Conventional	279 ± 159 kPa	18 ± 11 s	4.995 ± 3.002 ml	3:31 ± 1:32 min
Symani	215 ± 114 kPa	16.5 ± 12 s	4.579 ± 3.441 ml	7:39 ± 2:32 min

Table 1 Imaged are the mean values and the standard deviations (SD) for both operation procedures. The pressure maximum, the time until the pressure maximum was reached the filling volume as well as the operation time were calculated.

**Fig. 4:**
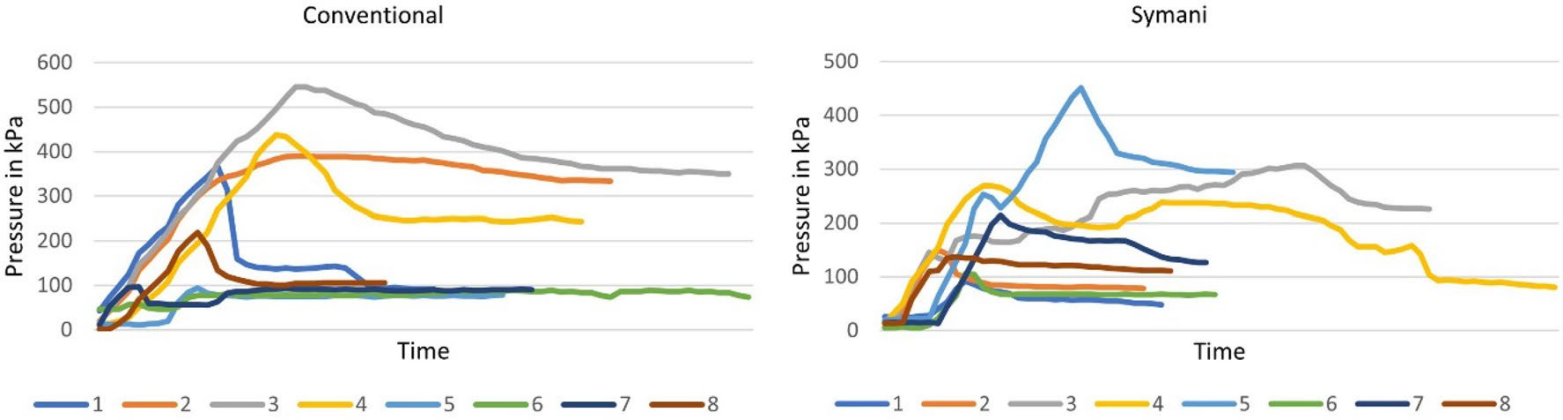
The pressure curve for all eight measurements using the conventional surgical method as well as the Symani

There were no significant differences for the pressure maximum (*p* = 0.528), for the time until the pressure maximum was reached (*p* = 0.528) or for the maximum filling volume (*p* = 0.674) (Fig. 3). The time needed for the suturing of the membrane was significantly longer in the robotic surgery group (*p* < 0.001). The pressure curve for each measurement has been shown in Fig. 4.

### SEM evaluation

**Fig. 5:**
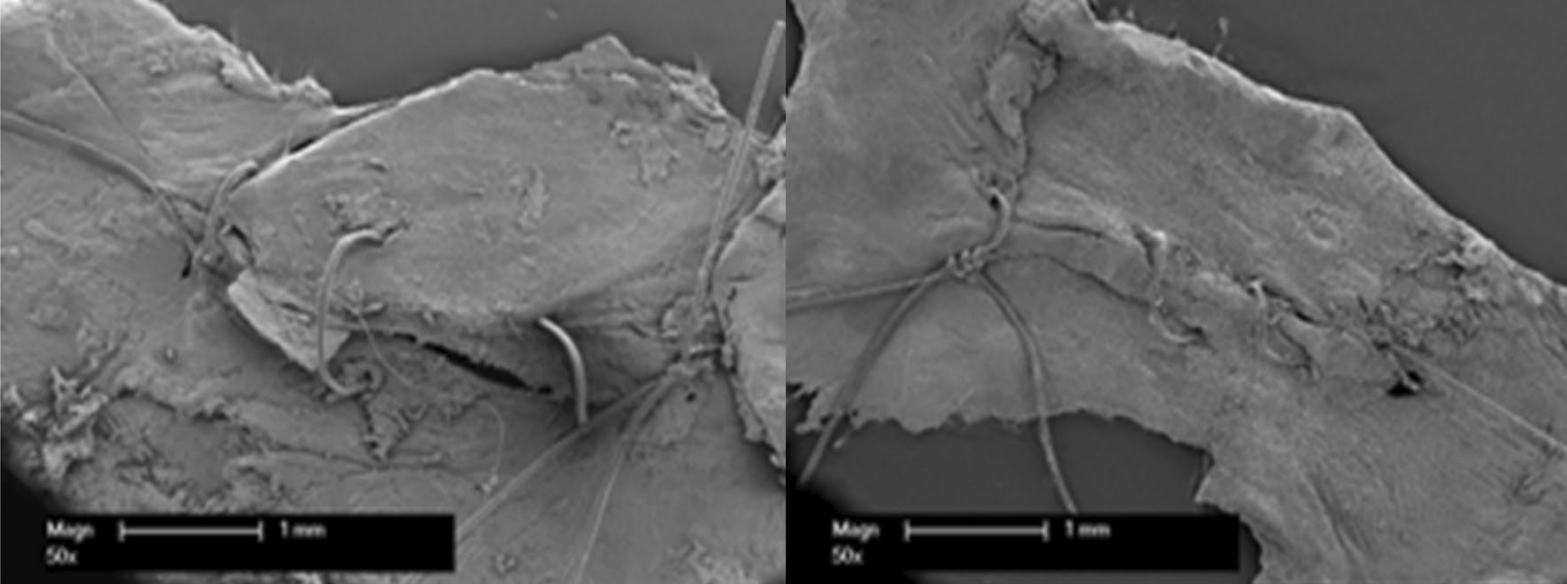
The scanning electron microscope revealed a better occlusion of the wound edges with robotic surgery (right) compared to the conventional technique

The quantification via scanning electron microscope (SEM) revealed a relatively better adaptation of the wound edges in the study group (Fig. 5).

## Discussion

Robotic surgery is a modern minimally invasive procedure that allows the reduction of morbidity through the minimally invasive approach with excellent optical representation of the surgical field [11]. In terms of functional outcomes, robotic surgery may show advantages in reducing morbidity in patients with cancer of the upper digestive and respiratory tracts [10]. It was originally developed for the disciplines such as general surgery and urology; however, it has gained popularity also in different surgical fields. Transoral robotic surgery (TORS) has been an established procedure since 2009 and the indication for robotic surgery with minimally invasive technique is increasing in the field of oral and maxillofacial surgery [9]. Moreover, TORS has advantages, such as that the robotic arms can be moved to various angles even within confined spaces of the maxillofacial region, as well as smaller and smaller operation procedures could be performed and operative complications could be prevented [12]. Initial concerns about robotic surgery for oral and maxillofacial surgery were about visualization, possible damage to vital structures, and the limited availability of effective instrumentation [6]. However, the system used for the study was developed specifically for the smallest anatomical structures in oral and maxillofacial surgery and plastic surgery, such as small anatomizes, to overcome the limits of manual precision and physiological tremor [7]. Despite the high fragility of the Schneiderian membrane, robotic surgery-assisted repair resulted in qualitatively better results compared to the conventional technique.

The management of intraoperative complications via using robotic systems has also become the subject of experimental studies. Kupferman et al. have used a robotic system for the repair of dura defects and reported successfully results [13]. Veleur et al. have reported the use of robot-assisted surgery for several categories of middle ear procedures including the repair of the tympanic membrane ruptures [14]. Perforation of the Schneiderian membrane is a common complication of sinus floor augmentation, with an incidence of 10.0–35.9% [15] and poses a great challenge for both dental and ENT clinicians and the efficiency of perforation’s closure plays a great role in the prognosis of the procedure, especially after grafting of the maxillary sinus [16]. Covering small perforations with resorbable membranes or fibrin glue and suturing are the main intraoperative solutions [17–19]. The results of the current study showed that robotic technology can be successfully implemented in Schneiderian membrane repair with efficient occlusion of the wound edges.

It is well known that the injuries or surgical interventions of the maxillary sinus could jeopardize the integrity of the Schneiderian membrane and air could penetrate the subcutaneous tissues. In some cases, if the air pressure in the maxillary sinus increases with sneezing, coughing or nose blowing, a traumatic subcutaneous emphysema could also occur [20]. Therefore, it is important to advise patients to avoid increasing the intraoral pressure after sinus-lift procedure [21]. Wu et al. have quantified sinus pressures along the skull base during sneezing and stated that the anterior nasal pressure was 3153.22 ± 957.36 Pa [22]. The results of the current study confirmed that the leakage after achieving the sinusoidal peak pressure was similar in both groups. Due to the limitations of the experimental model described herein, it should be mentioned that the pressure after reconstruction with both techniques remained significantly under the nasal pressure reached by sneezing, however, it is not possible to speculate on the efficiency of the performed reconstruction regarding the physiological limits.

Robotic surgery has been criticized for its expense (e.g., an estimated increase of $1500 to $2000 per patient) [23], however, the global surgical robots market size was valued at USD 4.4 billion in 2022 and is expected to expand at a compound annual growth rate of 18.0% from 2023 to 2030 [24]. MMI's complete Symani Surgical System including cart, console and NanoWrist instruments requires an investment of approximately 900,000 euro [25]. Due to their high costs and limited indication spectrum in dentistry, the use of robotic systems in daily dental practice is financially not feasible. Besides that, extensive training and intensive education are necessary to achieve optimal surgical results [26]. It also takes a long time to establish robotic surgery, although this procedure shows a steep learning curve for both young and experienced surgeons in many operation cases [27]. Barbon et al. already described the steep learning curve for anastomotic reconstructions of the Symani and stated that the system enables promising potential for limits in reconstructive microsurgery, such as reliably performing anastomoses on small blood and lymph vessels or on structures deeper in the body cavities [28]. Therefore, the feasibility regarding both the material costs and the steep learning curve should be taken into consideration before the deployment of the system in the field of dentistry.

Time pressure is the most common stressor on dental professionals’ performance [29]. Due to the patients’ demands regarding re-establishment of function, phonation and aesthetics within the shortest possible time, rapid rational concepts are increasingly becoming the preferred treatment option in the dental implantology practice [30]. It has been previously also demonstrated that the feasibility of performing precise micro sutures and anastomoses robotic procedures were longer in time using the Symani system, but showed greater precision compared to standard manual techniques [31]. Our results have also shown that, despite the high experience of the surgeon, the time needed for the suturing with robotic surgery was significantly longer compared to the conventional technique and could not offer an advantage for the dental clinicians, who are already working under time pressure.

## Conclusion

To the best of our knowledge, the technical feasibility of robot-assisted suturing of Schneiderian membrane laceration using the Symani Surgical System has been performed for the first time in the literature. Robotic suturing required a longer time to complete but was superior to the manual procedure regarding the SEM data collected. However, no differences considering the pressure resistance compared to the conventional repair could be observed. Regarding the material and training costs and limited indications spectrum, robotic surgery systems might not be a feasible option in daily dental practice yet.

### Supplementary Information

Below is the link to the electronic supplementary material.

Supplementary file1 (DOCX 12 KB)

Supplementary file2 (JPG 136 KB)

Supplementary file3 (MOV 446 KB)

## Data Availability

The data that support the findings of this study are available on request from the corresponding author (A.G.).
