# Supplementary figures and images for: A technical feasibility study on adaptation of a microsurgical robotic system to an intraoperative complication management in dental implantology: perforated Schneiderian membrane repair using Symani® Surgical System
Source: J Robot Surg. 2023 Oct 6;17(6):2861–7. doi: 10.1007/s11701-023-01721-9 (PMC10678809; doi:10.1007/s11701-023-01721-9)

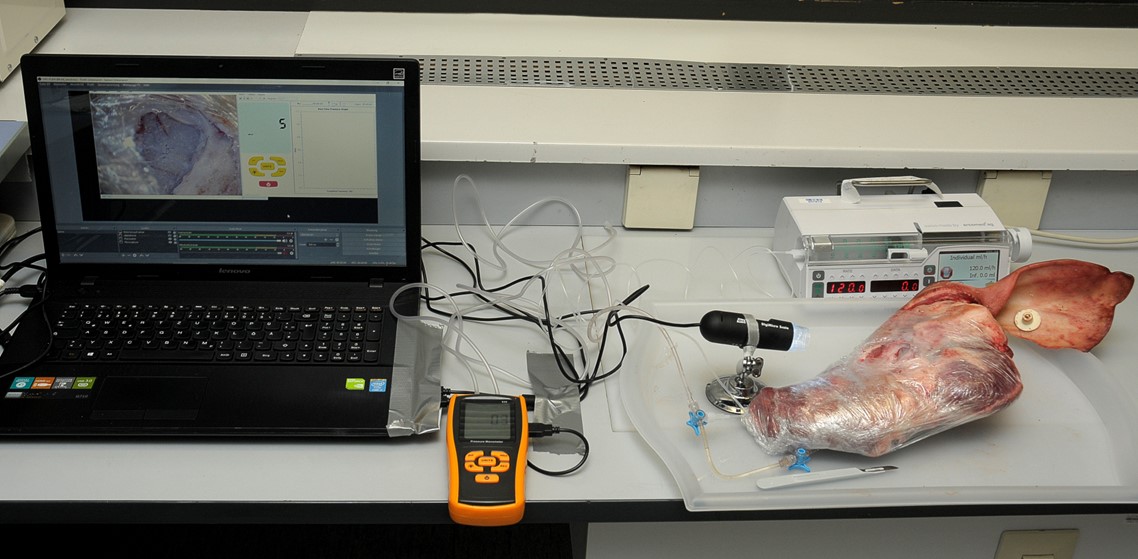

Supplement: Supplementary file 2 — Supplementary file2 (JPG 136 KB) [file 11701_2023_1721_MOESM2_ESM.jpg]
