# Supplementary material for: A technical feasibility study on adaptation of a microsurgical robotic system to an intraoperative complication management in dental implantology: perforated Schneiderian membrane repair using Symani® Surgical System
Source: J Robot Surg. 2023 Oct 6;17(6):2861–7. doi: 10.1007/s11701-023-01721-9 (PMC10678809; doi:10.1007/s11701-023-01721-9)
Supplement: Supplementary file 1 — Supplementary file1 (DOCX 12 KB) [file 11701_2023_1721_MOESM1_ESM.docx]

Dear Editor and Reviewers,

we added supplementary materials. A video of measuring the pressure, in which you can see the peak and the status of the „steady state“. Additionally a figure of the experimental setup. Maybe it would be useful for the readership for the part „Material and Methods“.
